# Un abcès péri-hépatique compliquant une perforation duodénale pris pour un abcès sous capsulaire du foie

**DOI:** 10.11604/pamj.2014.17.98.3949

**Published:** 2014-02-07

**Authors:** Toufik Joulali, Nabil Kanjaa

**Affiliations:** 1 Service de Réanimation Polyvalente A4, CHU Hassan II, Fès, Maroc

**Keywords:** Abcès hépatique, perforation duodénale, drainage, liver abscess, duodenal perforation, drainage

## Image en medicine

Les abcès du foie sont des amas de pus dans une cavité néo formée au dépens du tissue hépatique. L'origine biliare est la plus fréquente et ce sont le plus souvent des abcès multiples. L'orgine locale est souvent la consequence d'une perforation digestive avec une surinfection et une atteinte par contiguïté. L'évolution dépend de la rapidité du diagnostic et de la prise en charge ainsi que l'étiologie de l 'abcès avec une place prémordiale de la radiologie en matière de diagnostic et de prise en charge interventionnelle. Nous rapportons l 'observation de Mlle O.N âgée de 35 ans, suivie pour une pathologie ulcéreuse sous traitement avec une mauvaise observance thérapeutique, admise aux urgences pour la prise en charge de douleurs abdominales. L'examen à l 'admission a trouvé une patiente consciente avec un Glasgow à 15, fébrile à 39,7°, stable sur le plan hémodynamique et respiratoire avec une sensibilité abdominale plus accentuée au niveau de l'hypochondre droit. Le scanner abdominal a objective une perforation duodénale avec un abcès sous capsulaire du foie (A). L'exploration chirurgicale a montré une collection inter hépato-diaphragmatique sans fraction capsulaire ni anomalie du parenchymahépatique avec la mise en évidence de la perforation duodénale (B). La prise en charge a consisté à un drainage de la collection et aux sutures de la perforation avec des suites post-opératoires simples.

**Figure 1:**
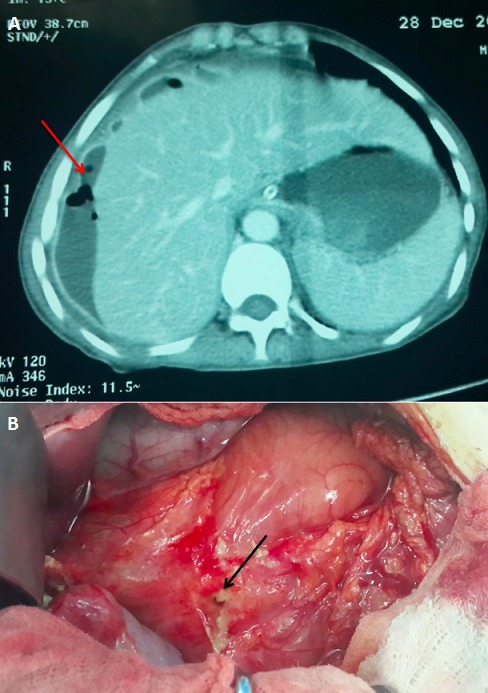
A) Coupe scannographique objectivant un abcès sous capsulaire du foie avec présence de bulles d'air; B) Image per-opératoire objectivant la perforation duodénale avec une partie du parenchyme hépatique sans anomalie

